# Craniofacial Morphology of Temporomandibular Disorder Patients with Different Disc Positions: Stratifying Features Based on Sex and Sagittal Skeletal Pattern

**DOI:** 10.3390/jcm12020652

**Published:** 2023-01-13

**Authors:** Chengxinyue Ye, Peidi Fan, Jie Zhang, Qiaoyu Cheng, Xin Xiong, Jun Wang

**Affiliations:** Department of Orthodontics, National Clinical Research Center for Oral Diseases, State Key Laboratory of Oral Diseases, West China Hospital of Stomatology, Sichuan University, Chengdu 610041, China

**Keywords:** temporomandibular disorders, disc displacement, magnetic resonance imaging, cephalometry, sex, sagittal skeletal pattern

## Abstract

Disc displacement (DD) appears in the majority of temporomandibular disorder (TMD) patients. The correlation between craniofacial morphology and different disc positions has been underlined, while the craniofacial morphological differences based on sex and sagittal skeletal pattern stratification have been insufficiently studied. In this study, 304 patients with TMD complaints were included and classified into normal position, disc displacement with reduction (DDwR) and disc displacement without reduction (DDwoR) groups according to magnetic resonance imaging. A total of 24 cephalometric measurements, covering the cranial base, vertical relationship, sagittal relationship, mandible position and morphology, and dental relationship, were assessed using lateral cephalograms. A stratified analysis was performed based on the sex and sagittal skeletal pattern. Overall, DD patients had distinctive craniofacial morphological features. The posterior cranial base length and mandibular arc angle were statistically different only in females, while the Y-axis angle, occlusal plane angle and sella nasion point A angle (SNA) might be unique features in males. Skeletal class II had the most statistically different measurements compared to the others. Differences in the Frankfort mandibular incisor angle (FMIA) and saddle angle were especially displayed in skeletal class III patients. The sex and skeletal sagittal pattern could affect the morphological differences in TMD patients with different disc positions.

## 1. Introduction

Temporomandibular disorders (TMDs) refer to a series of heterogeneous musculoskeletal disorders with diverse manifestations and pathogenies [1]. Pain, noises, deviated jaw movement and decreased mouth opening are most commonly complained symptoms of TMD patients [2]. With a high prevalence of 27–38% in the general population [3], TMDs have become an unneglectable thorny issue in clinical treatment. Articular disc displacement composes an important subtype of TMDs and is reported to be diagnosed in nearly 70% of the TMD patients [4,5]. The articular disc is normally located between the mandibular condyle and the articular eminence of the temporal bone and functions as a cushion between the two bone surfaces during jaw movement [6,7]. The alteration of the disc position, frequently in the anterior direction, is described as disc displacement (DD) [8]. In the diagnosis of DD, magnetic resonance imaging (MRI) is considered the gold standard. Through analyzing MRIs taken in closed-mouth and open-mouth positions, DD can be further classified into that with reduction (DDwR) and without reduction (DDwoR).

The mechanisms of DD remain controversial. Some studies have underlined the potential correlations between craniofacial morphology and different disc positions in TMD patients [9,10]. A retrusion and clockwise rotation of the mandible, smaller ramus height and decreased posterior facial height have been reported as remarkable morphological features in DD patients [11]. However, insufficient attention has been paid to potential confounding factors, such as age, sex, ancestry or environment, which are possible risk factors for TMDs as well as significant determinants in the formation of different craniofacial morphologies [12]. A higher prevalence of TMDs in females than males has been reported [13], while the mechanisms of such sex-related difference remain arguable. The role of malocclusion in the pathogenesis of TMDs is debatable [14]. It has been reported by some studies that dental malocclusion and transverse discrepancies should not be predictors of TMDs, but the retro-positioned mandibles might be distinct in joint disorder patients [14,15]. However, few studies have subdivided TMD patients based on skeletal pattern, and insufficient evidence has been provided in verifying the differences in skeletal class I, class II and class III DD patients. Contemporarily, a tailored and comprehensive treatment strategy has been suggested for TMD treatment, which has also enhanced the need for exquisite assessment methods and the precise subcategorization of patients [16,17]. Therefore, a study with a stratified comparative analysis is required in order to discriminate the stratifying features in different subgroups.

The aim of the present study is to depict the morphological features in DD patients by analyzing MRI in closed-mouth and open-mouth positions as well as lateral cephalograms, so as to provide more evidence for the pathology of DD in different sexes and sagittal skeletal patterns.

## 2. Materials and Methods

### 2.1. Study Population

This retrospective study was conducted on Chinese Han patients who visited the orthodontic department of our hospital between 1 January 2021 and 30 December 2021. Written informed consent was obtained from all patients. MRI and X-ray cephalometry were examined only when the patients had TMD complaints or clinical symptoms to avoid excessive examinations. All the procedures were conducted in accordance with the Declaration of Helsinki and approved by the Ethics Committee of West China Hospital of Stomatology, Sichuan University (No. WCH-SIRB-CT-2020-418, 31 December 2020). The inclusion criteria were as follows: (1) adult patients with permanent dentition, (2) with complaints of TMD symptoms, (3) with clear lateral cephalogram and MRI containing TMJ of both sides in closed-mouth and open-mouth positions. The exclusion criteria were as follows: (1) a history of tumor, maxillofacial deformity, trauma or craniofacial surgeries that might affect TMJ or craniofacial morphology; (2) a history of systemic diseases; (3) a history of orthodontic treatment or plastic surgery; (4) a history of TMD treatment.

### 2.2. Magnetic Resonance Imaging (MRI) Evaluation

**Figure 1:**
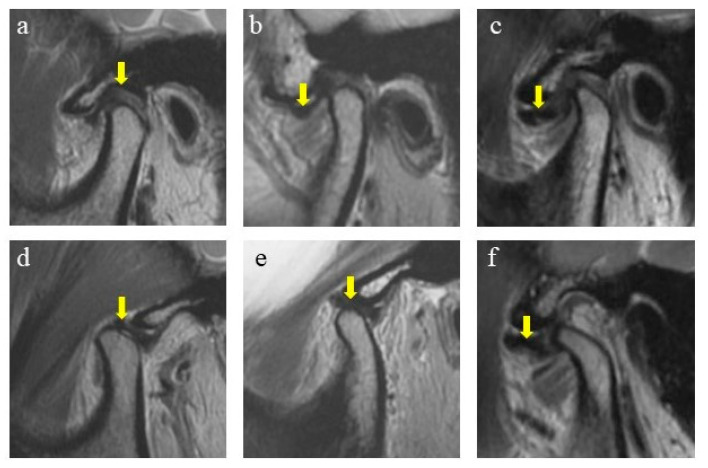
Diagnosis of disc displacements in MRIs. (**a**) Normal, in closed-mouth position; (**b**) DDwR, in closed-mouth position; (**c**) DDwoR, in closed-mouth position; (**d**) normal, in open-mouth position; (**e**) DDwR, in open-mouth position; (**f**) DDwoR, in open-mouth position. Arrow shows the position of the articular disc.

The MRIs were performed with a 1.5T MRI scanner (Philips, Amsterdam, The Netherlands) with a TMJ surface coil. To identify the disc position, T2-weighted imaging (T2WI) generated by a fast spin echo (FSE) sequence was examined. Parameter settings were: 2300 ms for repetition time; 45 ms for echo time; 3 mm for slice thickness; 0 mm for slice gap; 2 for number of excitations; 288 × 256 pixels for image matrix. The scanning included nonorthogonal sagittal and nonorthogonal coronal sections in the closed-mouth position and nonorthogonal sagittal sections in the open-mouth position. Patients assumed a supine position with the center of the coil positioned at a 10 mm anterior to the tragus. For the closed-mouth position, patients were instructed to occlude in the intercuspal position and hold the lips in a relaxed status. For the open-mouth position, patients were instructed to slowly reach maximum unassisted mouth opening (MMO), and then rubber pads with different thicknesses were applied to maintain the posture. MRI data were stored in DICOM format and observed with RadiAnt DICOM Viewer (Medixant, Poznan, Poland). The diagnosis of the subjects was categorized into 3 groups [18,19,20] (Figure 1). Two operators (C.Y. and X.X.) conducted the MRI diagnosis. The intra-observer and inter-observer reliability was verified before the study with a kappa coefficient >0.85.

#### 2.2.1. Normal Position

In the closed-mouth position, the intermediate zone of the articular disc was located between the posterior slope of the articular eminence and the anterior slope of the condyle head (defined as from 11:30 to 12:30 o’clock to the condyle head). In the open-mouth position, the intermediate zone was located between the top of the condyle head and the articular eminence. Subjects with articular discs of both sides in the normal position were assigned to the normal group.

#### 2.2.2. Disc Displacement with Reduction (DDwR)

In the closed-mouth position, the intermediate zone of the articular disc was anteriorly displaced relative to the anterior slope of the condyle head (defined as before 11:30 o’clock to the condyle head). In the open-mouth position, the intermediate zone was reduced to the top of the condyle head and articular eminence. Subjects diagnosed with bilateral DDwR or unilateral DDwR with the other in the normal position were assigned to the DDwR group.

#### 2.2.3. Disc Displacement without Reduction (DDwoR)

In the closed-mouth position, the intermediate zone of the articular disc was anteriorly displaced relative to the anterior slope of the condyle head. In the open-mouth position, the intermediate zone was still anteriorly displaced, which indicated no reduction. Subjects diagnosed with bilateral DDwoR or unilateral DDwoR, with or without DDwR for the other side, were assigned to the DDwoR group.

### 2.3. Cephalometric Evaluation

**Figure 2:**
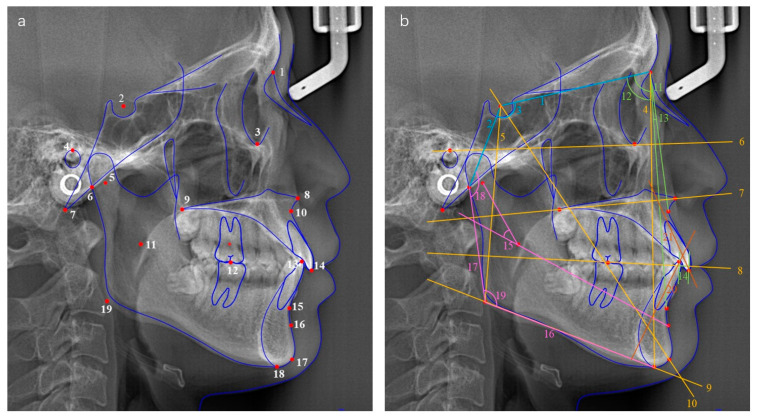
(**a**) Cephalometric landmarks used in the study. (1) N, nasion; (2) S, sella; (3) Or, orbitale; (4) P, porion; (5) Dc point; (6) Ar, articulare; (7) Ba, basion; (8) ANS, anterior nasal spine; (9) PNS, posterior nasal spine; (10) A point; (11) Xi point; (12) first molar occlusal point; (13) lower incisor occlusal point; (14) upper incisor occlusal point; (15) B point; (16) Pm point; (17) Gn, gnathion; (18) Me, menton; (19) Go, gonion. (**b**) Planes and angles used in the study. Cranial base (blue): (1) anterior cranial base length, S–N; (2) posterior cranial base length, S–Ar; (3) saddle angle. Vertical relationship (yellow): (4) anterior facial height, N–Me; (5) posterior facial height, S–Go; (6) Frankfort plane; (7) palatal plane; (8) occlusal plane; (9) mandibular plane; (10) Y-axis. Sagittal relationship (green): (11) SNA; (12) SNB; (13) ANB; (14) Wits. Mandible position and morphology (pink): (15) mandibular arc angle, Dc–Xi–Pm; (16) mandibular body length; (17) ramus height; (18) articular angle; (19) gonial angle. Dental relationship (orange): (20) LI axis; (21) UI axis.

The cephalograms were shot by using an X-ray scanner (Morita, Osaka, Japan) with the standardized technique. Each patient was instructed to assume a natural head position and occlude in ICP without swallowing or chewing. Digital cephalograms were anonymized and then traced using Uceph software (version 961, Chengdu, China). The Frankfort horizontal plane (FH plane) was considered as the reference plane. A total of 24 measurements (Figure 2 and Table 1) were performed to evaluate the cranial base, vertical relationship, sagittal relationship, mandible position and morphology, and dental relationship, respectively. Sagittal skeletal pattern categorization was based on the ANB angle as follows: (1) skeletal class I (ANB < 1°), (2) skeletal class II (1° ≤ ANB ≤ 5°) and (3) skeletal class III (ANB > 5°). Two operators (C.Y. and X.X.) conducted the cephalometric tracing. The intra-observer and inter-observer reliability was verified before the study with a kappa coefficient >0.80 for all the measurement items.

### 2.4. Statistical Analysis

Statistical analyses were performed with Empowerstats (http://www.empowerstats.com, X&Y Solutions, Inc., Boston, MA, USA, assessed on 9 February 2022). Quantitative data are presented as mean ± standard deviation. Categorial data are presented as frequency and constituent ratio. The differences in quantitative data were evaluated through one-way analysis of variance (ANOVA). The differences in categorical variables were evaluated through R × C Chi-square test. Post-hoc comparisons were performed using the Student–Newman–Keuls (SNK) method. A stratified analysis was performed based on sex and sagittal skeletal pattern with the same statistical methods. An α error of 0.05 and a statistical power (1-β) of 80% was set for the statistical analysis. Based on a size effect estimated by posterior facial height, the required minimum sample size was set at 24 for each group.

## 3. Results

### 3.1. Demographic Information

**Figure 3:**
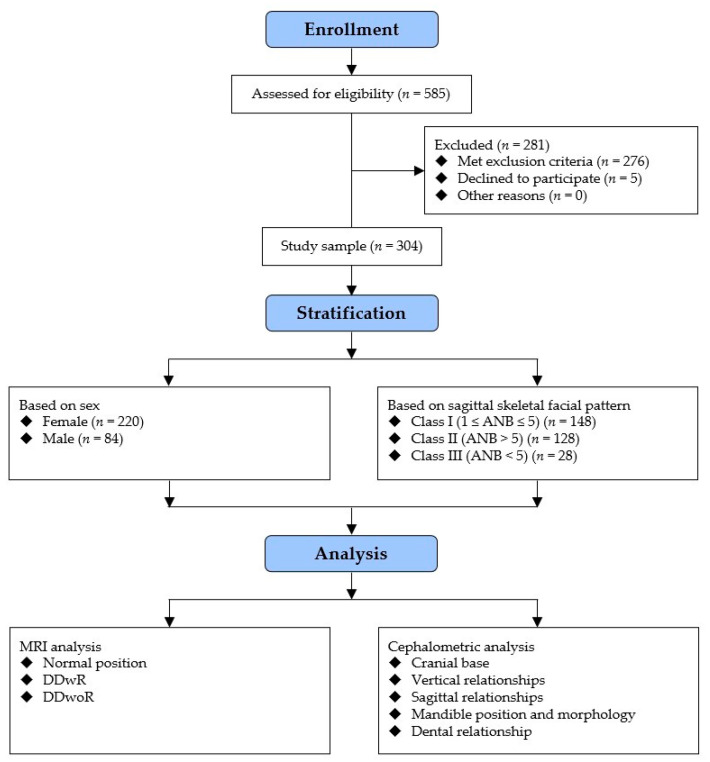
Flow diagram of the study.

The study enrolled 304 Chinese Han adults with TMDs (Figure 3), among whom 90 were categorized into the normal group, 99 into the DDwR group, and 115 into the DDwoR group (Table 2). The average age of the subjects was 30.8 ± 10.3 with no significant difference shown among the three groups (*p* = 0.131). Regarding sex, the DDwR and DDwoR groups showed higher proportions of females than the normal group (*p* < 0.001). The constituent ratio of different sagittal skeletal patterns in the three groups presented no significant difference (*p* = 0.126), while there was a slight rise in the proportion of class II in the DDwoR group.

### 3.2. Overall Analysis

**Figure 4:**
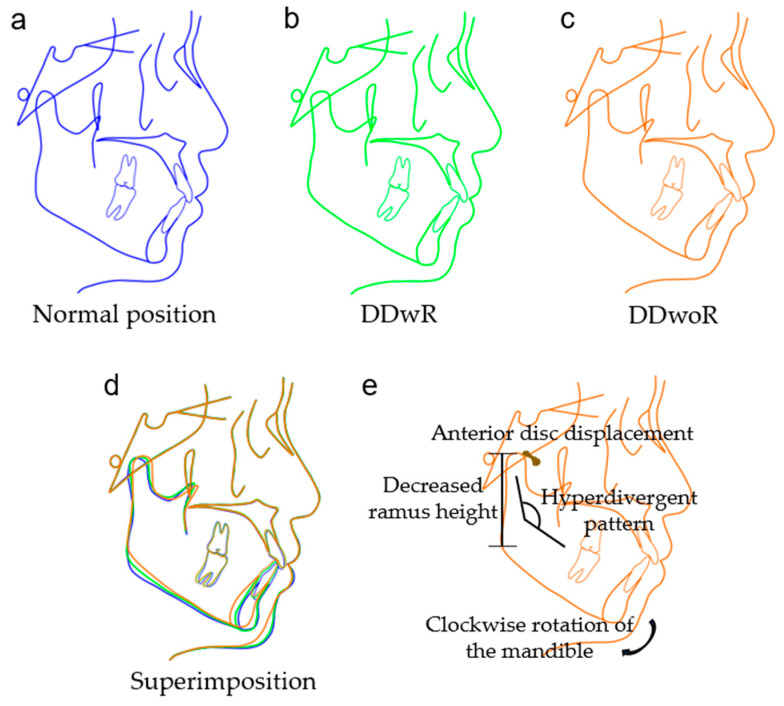
Schematic figures showing morphological characteristics of TMD patients with different disc positions. (**a**) Normal group (blue); (**b**) DDwR group (green); (**c**) DDwoR group (orange); (**d**) superimposition of the three groups; (**e**) schematic illustration of the morphological changes in the DDwoR group compared with the normal group.

A total of 12 measurements showed statistical differences (Table 3 and Figure 4), including the posterior cranial base length (*p* = 0.013), posterior facial height (*p* < 0.001), facial height index (*p* < 0.001), Y-axis angle (*p* = 0.036), mandibular plane angle (*p* < 0.001), occlusal plane angle (*p* = 0.048), SNA (*p* = 0.006), SNB (*p* < 0.001), mandibular arc angle (*p* = 0.003), ramus height (*p* < 0.001), gonial angle (*p* = 0.034) and IMPA (*p* = 0.012).

### 3.3. Stratified Analysis Based on Sex

Among the 220 female subjects studied, differences in posterior cranial base length (*p* = 0.013), posterior facial height (*p* < 0.001), facial height index (*p* < 0.001), mandibular plane angle (*p* = 0.013), SNB (*p* = 0.007), mandibular arc angle (*p* = 0.004) and ramus height (*p* = 0.001) were statistically different (Table 4 and Table S1). The results of the 84 male subjects were subtly different from the females. Measurements with statistical differences included posterior facial height (*p* = 0.047), facial height index (*p* = 0.034), Y-axis angle (*p* = 0.012), occlusal plane angle (*p* = 0.050), SNA (*p* = 0.003) and SNB (*p* = 0.005).

### 3.4. Stratified Analysis Based on Sagittal Skeletal Pattern

Among the 148 class I subjects, measurements with statistical differences consisted of the posterior facial height (*p* = 0.009), facial height index (*p* = 0.012), ramus height (*p* = 0.011) and IMPA (*p* = 0.021) (Table 5 and Table S2). Among the 128 class II subjects, significant differences were observed in 10 measurements including the posterior cranial base length (*p* = 0.018), posterior facial height (*p* < 0.001), facial height index (*p* = 0.001), mandibular plane angle (*p* = 0.008), occlusal plane angle (*p* = 0.037), SNA (*p* = 0.020), SNB (*p* = 0.025), mandibular arc angle (*p* = 0.009), ramus height (*p* < 0.001), and IMPA (*p* = 0.012). Among the 28 class III subjects, significant differences were seen in the saddle angle (*p* = 0.042), SNA (*p* = 0.021), IMPA (*p* = 0.034) and FMIA (*p* = 0.033).

## 4. Discussion

The present study demonstrated that the differences in craniofacial morphology were associated with different disc positions. Only adult patients were included in order to avoid the influence of growth on the measurements. A total of 70.39% of the TMD patients included in the study were diagnosed with DD, which coincided with the proportions reported in the previous literature. A higher susceptibility to DD in females was suggested, with a significantly wider gap in the DDwR group and the DDwoR group. A slight increase in the proportion of skeletal class II patients with DDwoR could be observed, although the difference was not statistically significant.

Among the cranial base measurements, the posterior cranial base length was significantly smaller in the DD groups than in the normal group. This trend was common in all the stratified analyses, although in some subgroups no significant differences were present due to a limited sample volume. The posterior cranial base length, defined by measuring the sella to the articulare or basion, is closely related to skeletal pattern in terms of mandible position. The relationship between the cranial base and sagittal discrepancies has been widely discussed, but no consensus has yet been reached [21,22,23]. The smaller posterior cranial base possibly suggested a clockwise rotation of the mandible and a skeletal class II inclination, which was in line with the measurements for skeletal relationships in this study. The anterior cranial base length and saddle angle exhibited no significant difference among the three groups in the overall analysis. As for the stratified analysis, interestingly, a significant difference existed in the measurement for saddle angle in the class III subgroup.

Among the vertical relationship measurements, an insufficient posterior facial height and steeper mandibular plane angle were observed in the DD groups, in line with the previous literature [11]. The trend was more pronounced in the DDwoR group compared with the DDwR group. A clockwise rotation of the mandible, obtuse growth pattern of the mandible and suppressed ramus growth were possible causes of the unique manifestation. However, posterior facial height and mandibular plane angle were not significant indicators for DD in class III patients. A smaller Y-axis angle was shown in the DDwR group but not in the DDwoR group. Few studies have covered the Y-axis angle in measurements. Gidarakou et al. reported a larger Y-axis angle in symptomatic female patients with bilateral DDwoR in comparison with asymptomatic female volunteers [24]. Interestingly, the Y-axis angle was found to be statistically different in males but not in females, indicating that it might be a unique indicator for male TMD patients. A larger occlusal plane angle in the DDwoR group than in the DDwR group was found, especially in the male subgroup and the class II subgroup. Many previous studies focusing on female subjects reported no significance in the occlusal plane angle, which coincided with the findings in this study [25].

Among the sagittal relationship measurements, both the SNA and SNB were significantly smaller in the DDwoR group than in the DDwR and normal groups, indicating a retrusion of both the maxillae and mandible. A decrease in the SNB has been the consensus in most of the previous studies, but results on the SNA have varied [25,26,27]. A longitudinal study with a follow-up time of over three years by Carlos Flores-Mir et al. pointed out that TMJ disc abnormality was associated with reduced forward growth of the maxillae and mandible, which supported the results in this study [28]. The results of the stratified analysis might provide an explanation for the controversy surrounding the SNA, as this trend especially existed in males and was therefore not shown in the studies with only females included. Notably, the SNA and SNB use the cranial base as the reference to appraise the positions of the jaws and therefore the relationship between DD and the cranial base might influence the SNA and SNB angle. Similarly, the measurements of Wits might as well be misinterpreted owing to the change in the occlusal plane. It was suggested that more references for assessments, such as the bisector of the palatal plane to the mandibular plane angle, should be applied in the sagittal relationship appraisal [29].

Among the mandible position and morphology measurements, differences were observed in the mandibular arc angle, ramus height and gonial angle. The findings in this study suggested the mandibular body length remained while the ramus height descended. One main theory for the ramus height decrease was that the ramus would undergo osseous changes and compromise its height under abnormal mechanical loading caused by DD, as abundant studies have revealed the effectiveness of mechanical forces in affecting bone growth and remodeling [30,31,32]. An obtuse mandibular growth pattern in the DDwoR group was exhibited by a smaller mandibular arc angle and larger gonial angle. The rotational change in the mandible caused by the shortening ramus height and obtuse mandibular growth pattern might explain the hyperdivergent facial profile of DD patients. Differences in mandible position and morphology were especially significant in female and class II subgroups.

Among the dental relationship measurements, only the IMPA was statistically different and the decreasing trend of the IMPA existed in all subgroups. The FMIA was similar in class I and II, which indicated that the difference in IMPA might be attributed to the distinction of the FMA. Interestingly, the FMIA was significantly larger in the skeletal class III group. Moon et al. reported a similar change in the FMIA in 66 women with an oversized mandible and TMJ internal disarrangement [25]. The decreased FMIA indicated that lingual-inclined lower incisors, as a compensatory change to combat the skeletal class III relationship, might be a unique feature in class III patients with DD.

TMDs are reported to appear remarkably more frequent and severe in females than in males [33]. The sex-related difference is an intriguing issue from a biopsychosocial view. Different muscle fiber compositions and strengths could influence the stabilization of the articular disc [34]. Estrogen metabolism might also greatly contribute to the osseous changes of the condyle and enhance pain sensibility [35,36]. As they are closely related to abnormal oral parafunctions such as grinding and clinching, or sleep disorders, some psychological and social indicators might be unneglectable topics in studying these differences [37]. In this study, sex-related morphological changes in DD were significant. Mandibular morphological measurements including mandibular arc angle and ramus height were highly specific in females, indicating the crucial effects on mandibular bone growth and remodeling. In males, the Y-axis angle, occlusal plane angle and SNA were specific, concerning the growth direction of the maxillae and mandible.

Several studies have presented a debated view on the susceptibility to TMDs in different skeletal malocclusions [38]. Studies by Abrahamsson et al. and Paunonen et al. both reported that orthognathic surgeries could have a positive effect on TMDs, reflecting the correlation between skeletal malocclusion and TMDs [39,40]. Skeletal facial patterns greatly influence the osseous and muscular transmission of occlusal force in the stomatognathic system, therefore influencing the adaptive remodeling of the temporomandibular joint [41]. The results of this study agreed with the viewpoint that sagittal skeletal patterns were related with DD, as DD patients of different sagittal skeletal patterns had different indicators of craniofacial morphology. Class II patients exhibited the most significant indicators. A clockwise rotation of the mandible, reflected by a larger mandibular plane angle, larger occlusal angle and smaller SNB, is the typical feature of class II DD patients. In class III patients, the saddle angle and FMIA are the unique indicators. The FMIA, which suggested compensatory lingual-inclined lower incisors, drew more attention to the occlusal interference in class III DD patients.

The main purpose of this study was the stratified analysis design based on sex and sagittal skeletal pattern, rendering the discovery of statistically different parameters in different subgroups. Relatively more cephalometric measurements than previous studies were covered in this study in order to give clinicians and researchers a comprehensive view on the craniofacial morphology of DD patients. A limitation of this study is the relatively small sample size of class III subjects owing to imbalanced proportions of visiting patients. Owing to the study design, which is a monocentric cross-sectional study, the generalizability of the findings might be impaired and causal relationships between DD and morphological differences remain unreached. Multicentric and longitudinal study design is anticipated for the verification of the conclusions. Some factors including local or systemic comorbidities, medication and socio-demographic characteristics might represent a confounding source that influences disc positions, where further stratification analysis would be interesting. As the lateral cephalograms were unable to exhibit craniofacial asymmetry, the current study did not distinguish lateral from bilateral lesions. The development of three-dimensional cephalometric methods might help discover asymmetric morphological features in lateral lesions.

## 5. Conclusions

TMD patients with different disc positions had distinctive craniofacial morphology. All of the five aspects studied in this study showed significant differences, namely the cranial base, vertical relationship, sagittal relationship, mandible position and morphology, and dental relationship. The sex and skeletal sagittal pattern could influence the pattern of difference. Mandibular morphological measurements were highly remarkable in females, while the growth direction of the maxillae and mandible was especially concerned in males. Clockwise rotation is a notable feature in class II patients. In class III patients, incisal interference owing to the compensatory change might be especially cautioned. These measurements provided evidence for the sex-related and sagittal-skeletal-pattern-related differences in DD mechanism and might assist in the detection and discrimination of potential DD patients in clinical settings.

## Figures and Tables

**Table 1:** Cephalometric measurements and definitions.

Measurements	Definitions
1. Cranial base	
Anterior cranial base length (mm)	S–N, distance between sella (S) and nasion (N)
Posterior cranial base length (mm)	S–Ar, distance between sella (S) and articulare (Ar)
Saddle angle (°)	Angle formed by S–Ar line and S–N plane
2. Vertical relationship	
Anterior facial height (mm)	N–Me, distance between nasion (N) and menton (Me)
Posterior facial height (mm)	S–Go, distance between sella (S) and Gonion (Go)
Facial height index (%)	Posterior facial height/anterior facial height × 100%
Y-axis angle (°)	Angle formed by S–Gn line and FH plane
Palatal plane angle (°)	PP–FH, angle formed by PP plane and FH plane
Mandibular plane angle (FMA) (°)	MP–FH, angle formed by MP plane and FH plane
Occlusal plane angle (°)	OP–FH, angle formed by OP plane and FH plane
3. Sagittal relationship	
Sella–nasion–point A angle (SNA) (°)	Angle formed by nasion–A line and SN plane
Sella–nasion–point B angle (SNB) (°)	Angle formed by nasion–B line and SN plane
Point A–nasion–point B angle (ANB) (°)	Angle formed by nasion–A line and nasion-B line
Wits (mm)	Distance between vertical lines from A point and B point to OP plane
4. Mandible position and morphology	
Mandibular Arc (°)	Dc–Xi–Pm, angle formed by Dc–Xi line and Xi–Pm line
Mandibular body length (mm)	Go–Me, distance between gonion (Go) and menton (Me)
Ramus height (mm)	Ar–Go, distance between articulare (Ar) and gonion (Go)
Articular angle (°)	S–Ar–Go, angle formed by S–Ar line and Ar–Go line
Gonial angle (°)	Ar–Go–Me, angle formed by Ar–Go line and MP plane
5. Dental relationship	
Interincisal Angle (°)	U1–L1, angle formed by the long axes of the upper and lower incisor
Incisor mandibular plane angle (IMPA) (°)	L1–MP, angle formed by the long axes of the lower incisors and MP plane
Frankfort mandibular incisor angle (FMIA) (°)	L1–FH, angle formed by the long axes of the lower incisors and FH plane
Overbite (mm)	Horizontal distance between the upper and lower incisal edges
Overjet (mm)	Vertical distance between the upper and lower incisal edges

**Table 2:** Demographic Information.

		Overall (*n* = 304)	Normal (*n* = 90)	DDwR (*n* = 99)	DDwoR (*n* = 115)	*p*-Value
Average age (year)		30.8 ± 10.3	32.67 ± 11.14	30.20 ± 9.72	29.94 ± 10.07	0.131
Age range (year)		18.1~71.8	18.1~71.8	18.3~62.7	18.3~68.2	
Sex categorial	Female	220 (72.36%)	51 (56.67%)	79 (79.80%)	90 (78.26%)	<0.001 **
	Male	84 (27.63)	39 (43.33%)	20 (20.20%)	25 (21.74%)	
Sagittal skeletal pattern categorial	Class I (female/male)	148 (48.68%) (100/48)	49 (54.44%) (27/22)	51 (51.52%) (37/14)	48 (41.74%) (36/12)	0.126
	Class II (female/male)	128 (42.11%) (102/26)	33 (36.67%) (20/13)	36 (36.36%) (33/3)	59 (51.30%) (49/10)	
	Class III (female/male)	28 (9.21%) (18/10)	8 (8.89%) (4/4)	12 (12.12%) (9/3)	8 (6.96%) (5/3)	

Quantitative data presented as mean ± SD; Categorial data presented as frequency (constituent ratio); ** *p* < 0.01.

**Table 3:** Comparison of cephalometric measurements in normal, DDwR and DDwoR groups.

	Overall	Normal	DDwR	DDwoR	*p*-Value	Post-Hoc
1. Cranial base						
Anterior cranial base length (mm)	62.75 ± 3.30	63.22 ± 3.36	62.89 ± 3.20	62.27 ± 3.30	0.109	
Posterior cranial base length (mm)	33.41 ± 3.49	34.57 ± 3.67	33.42 ± 2.92	32.49 ± 3.55	<0.001 **	1 > 2, 3
Saddle angle (°)	124.82 ± 5.24	125.24 ± 5.69	124.01 ± 4.94	125.20 ± 5.10	0.171	
2. Vertical relationship						
Anterior facial height (mm)	115.62 ± 6.87	116.54 ± 6.69	114.80 ± 7.29	115.60 ± 6.61	0.223	
Posterior facial height (mm)	77.61 ± 6.82	80.56 ± 7.08	77.56 ± 5.61	75.35 ± 6.73	<0.001 **	1 > 2 > 3
Facial height index (%)	67.19 ± 5.20	69.17 ± 5.19	67.67 ± 4.58	65.23 ± 5.07	<0.001 **	1 > 2 > 3
Y-axis angle (°)	61.08 ± 3.60	61.25 ± 3.47	60.34 ± 3.51	61.58 ± 3.70	0.036 *	2 < 3
Palatal plane angle (°)	0.01 ± 2.72	−0.20 ± 2.87	−0.05 ± 2.68	0.22 ± 2.64	0.522	
FMA (°)	24.20 ± 5.81	22.89 ± 6.12	23.48 ± 5.43	25.85 ± 5.54	<0.001 **	1, 2 < 3
Occlusal plane angle (°)	8.31 ± 4.15	7.99 ± 3.94	7.74 ± 3.78	9.05 ± 4.51	0.048 *	NS
3. Sagittal relationship						
SNA (°)	82.73 ± 3.56	83.39 ± 3.67	83.09 ± 3.24	81.91 ± 3.60	0.006 **	1, 2 > 3
SNB (°)	78.32 ± 3.98	78.96 ± 4.21	79.20 ± 3.62	77.07 ± 3.80	<0.001 **	1, 2 > 3
ANB (°)	4.41 ± 2.90	4.43 ± 2.77	3.89 ± 2.95	4.85 ± 2.90	0.053	
Wits (mm)	0.50 ± 3.89	0.76 ± 3.66	−0.02 ± 3.86	0.76 ± 4.07	0.264	
4. Mandible position and morphology						
Mandibular arc angle (°)	37.40 ± 5.35	38.71 ± 5.40	37.64 ± 4.71	36.17 ± 5.61	0.003 **	1, 2 > 3
Mandibular body length (mm)	69.60 ± 5.02	70.26 ± 4.98	70.01 ± 4.75	68.72 ± 5.19	0.055	
Ramus height (mm)	46.81 ± 5.02	48.90 ± 5.20	46.75 ± 4.36	45.23 ± 4.86	<0.001 **	1 > 2 > 3
Articular angle (°)	151.20 ± 6.89	150.09 ± 6.83	151.01 ± 6.22	152.23 ± 7.36	0.082	
Gonial angle (°)	117.51 ± 6.42	116.16 ± 6.63	117.58 ± 6.01	118.51 ± 6.48	0.034 *	1 < 3
5. Dental relationship						
Interincisal angle (°)	127.25 ± 13.20	126.46 ± 13.38	127.10 ± 14.12	127.98 ± 12.29	0.711	
IMPA (°)	96.71 ± 8.30	98.74 ± 8.10	96.54 ± 8.75	95.28 ± 7.80	0.012 *	1 > 3
FMIA (°)	59.09 ± 9.32	58.39 ± 8.26	59.99 ± 9.93	58.87 ± 9.59	0.476	
Overbite (mm)	2.57 ± 2.22	2.57 ± 2.34	2.52 ± 1.82	2.62 ± 2.45	0.952	
Overjet (mm)	4.02 ± 1.94	3.99 ± 2.37	3.78 ± 1.80	4.26 ± 1.66	0.200	

Data presented as mean ± SD; NS no significance, * *p*-value < 0.05, ** *p* < 0.01.

**Table 4:** Stratified analysis based on sex.

	Overall	Normal	DDwR	DDwoR	*p*-Value	Post-Hoc
**Female**						
1. Cranial base						
Posterior cranial base length (mm)	32.32 ± 2.87	32.82 ± 2.91	32.78 ± 2.55	31.64 ± 3.00	0.013 *	1, 2 > 3
2. Vertical relationship						
Posterior facial height (mm)	75.11 ± 5.11	76.40 ± 4.76	76.26 ± 4.75	73.37 ± 5.17	<0.001 **	1, 2 > 3
Facial height index (%)	66.21 ± 4.66	67.48 ± 4.49	67.31 ± 4.53	64.53 ± 4.39	<0.001 **	1, 2 > 3
FMA (°)	24.67 ± 5.34	23.84 ± 5.56	23.76 ± 5.39	25.94 ± 4.96	0.013 *	1, 2 < 3
3. Sagittal relationship						
SNB (°)	77.98 ± 3.74	78.03 ± 3.74	78.92 ± 3.52	77.13 ± 3.76	0.007 **	1, 2 > 3
4. Mandible position and morphology						
Mandibular arc angle (°)	37.14 ± 5.22	38.48 ± 5.42	37.82 ± 4.77	35.79 ± 5.22	0.004 **	1, 2 > 3
Ramus height (mm)	45.21 ± 3.97	46.24 ± 3.69	45.93 ± 3.72	44.00 ± 4.04	0.001 **	1, 2 > 3
5. Dental relationship						
None						
**Male**						
1. Cranial Base						
None						
2. Vertical relationship						
Posterior facial height (mm)	84.18 ± 6.36	86.01 ± 5.82	82.72 ± 5.90	82.49 ± 6.97	0.047 *	NS
Facial height index (%)	69.75 ± 5.68	71.38 ± 5.25	69.11 ± 4.61	67.72 ± 6.48	0.034 *	1 > 3
Y-axis angle (°)	61.22 ± 3.95	61.13 ± 3.60	59.38 ± 3.52	62.84 ± 4.24	0.012 *	2 < 3
Occlusal plane angle (°)	8.02 ± 4.83	7.43 ± 4.15	6.76 ± 4.34	9.95 ± 5.72	0.050	2 < 3
3. Sagittal relationship						
SNA (°)	83.20 ± 3.96	84.52 ± 3.83	83.15 ± 3.52	81.18 ± 3.75	0.003 **	1 > 3
SNB (°)	79.21 ± 4.47	80.19 ± 4.51	80.30 ± 3.91	76.82 ± 4.03	0.005 **	1, 2 > 3
4. Mandible position and morphology						
None						
5. Dental relationship						
None						

Data presented as mean ± SD; NS no significance, * *p*-value < 0.05, ** *p* < 0.01.

**Table 5:** Stratified analysis based on sagittal skeletal pattern.

	Overall	Normal	DDwR	DDwoR	*p*-Value	Post-Hoc
**Skeletal Class I**						
1. Cranial base						
None						
2. Vertical relationship						
Posterior facial height (mm)	78.77 ± 6.68	80.97 ± 7.05	78.41 ± 5.61	76.90 ± 6.84	0.009 **	1 > 3
Facial height index (%)	68.34 ± 4.78	69.80 ± 4.94	68.25 ± 4.36	66.94 ± 4.70	0.012 *	1 > 3
3. Sagittal relationship						
None						
4. Mandible position and morphology						
Ramus height (mm)	47.70 ± 4.91	49.32 ± 5.47	47.37 ± 4.33	46.40 ± 4.52	0.011 *	1 > 2, 3
5. Dental relationship						
IMPA (°)	95.53 ± 7.43	97.37 ± 8.07	95.89 ± 7.74	93.27 ± 5.78	0.021 *	1 > 3
**Skeletal Class II**						
1. Cranial base						
Posterior cranial base length (mm)	32.82 ± 3.50	34.05 ± 4.15	33.12 ± 2.45	31.95 ± 3.47	0.018 *	1 > 3
2. Vertical relationship						
Posterior facial height (mm)	76.08 ± 6.44	79.49 ± 7.02	76.43 ± 4.56	73.95 ± 6.31	<0.001 **	1 > 2, 3
Facial height index (%)	65.54 ± 0.50	67.52 ± 4.66	66.48 ± 4.62	63.86 ± 4.94	0.001 **	1, 2 > 3
FMA (°)	26.26 ± 5.69	24.62 ± 5.30	25.06 ± 5.75	27.92 ± 5.50	0.008 **	1, 2 < 3
Occlusal plane angle (°)	9.63 ± 3.95	8.98 ± 2.96	8.66 ± 3.79	10.59 ± 4.35	0.037 *	NS
3. Sagittal relationship						
SNA (°)	83.59 ± 3.51	84.38 ± 3.58	84.32 ± 2.93	82.71 ± 3.64	0.020 *	NS
SNB (°)	76.64 ± 3.71	77.40 ± 3.83	77.51 ± 3.05	75.68 ± 3.84	0.025 *	NS
4. Mandible position and morphology						
Mandibular arc angle (°)	36.34 ± 4.94	37.86 ± 5.42	37.27 ± 3.88	34.92 ± 4.91	0.009 **	1, 2 > 3
Ramus height (mm)	45.53 ± 4.62	47.87 ± 4.33	45.86 ± 3.84	44.02 ± 4.68	<0.001 **	1 > 2, 3
5. Dental relationship						
IMPA (°)	100.29 ± 6.96	102.34 ± 6.18	100.91 ± 7.51	98.77 ± 6.78	0.049 *	NS
**Skeletal Class III**						
1. Cranial base						
Saddle angle (°)	123.69 ± 5.61	125.74 ± 5.65	120.68 ± 4.78	126.16 ± 5.13	0.042 *	NS
2. Vertical relationship						
None						
3. Sagittal relationship						
SNA (°)	80.93 ± 3.34	82.20 ± 3.07	81.86 ± 2.95	78.26 ± 2.93	0.021 *	1, 2 > 3
4. Mandible position and morphology						
None						
5. Dental relationship						
IMPA (°)	86.61 ± 8.44	92.25 ± 9.49	86.18 ± 6.99	81.64 ± 6.59	0.034 *	1 > 3
FMIA (°)	71.30 ± 7.03	66.43 ± 7.98	71.94 ± 6.19	75.21 ± 4.60	0.033 *	1 < 3

Data presented as mean ± SD; NS no significance, * *p*-value < 0.05, ** *p* < 0.01.

## Data Availability

The data presented in this study are available on request from the corresponding author upon reasonable request.

## Supplementary Materials

The following supporting information can be downloaded at: https://www.mdpi.com/article/10.3390/jcm12020652/s1, Table S1: Stratified analysis based on sex; Table S2: Stratified analysis based on sagittal skeletal pattern.
