# Supplementary material for: Craniofacial Morphology of Temporomandibular Disorder Patients with Different Disc Positions: Stratifying Features Based on Sex and Sagittal Skeletal Pattern
Source: J Clin Med. 2023 Jan 13;12(2):652. doi: 10.3390/jcm12020652 (PMC9860960; doi:10.3390/jcm12020652)
Supplement: Supplementary file 1 [file jcm-12-00652-s001.zip › jcm-2118857-supplementary.pdf]

Table S1. Stratified analysis based on sex

		Overall	Normal	DDwR	DDwoR	*P* value	Post-hoc
1. Cranial Base							
Anterior cranial base length (mm)	Female	61.59±2.68	61.32±2.56	62.10±2.89	61.28±2.51	0.102	
	Male	65.82±2.80	65.71±2.58	65.99±2.45	65.84±3.43	0.936	
Posterior cranial base length (mm)	Female	32.32±2.87	32.82±2.91	32.78±2.55	31.64±3.00	0.013*	1, 2 > 3
	Male	36.24±3.40	36.85±3.31	35.93±3.00	35.55±3.77	0.295	
Saddle angle (°)	Female	124.88±5.28	125.83±5.62	124.12±4.92	125.01±5.35	0.191	
	Male	124.67±5.18	124.47±5.76	123.57±5.15	124.67±5.18	0.323	
2. Vertical relationship							
Anterior facial height (mm)	Female	113.60±5.90	113.36±4.97	113.53±6.88	113.81±5.49	0.904	
	Male	120.90±6.44	120.69±6.40	119.85±6.78	122.06±6.33	0.507	
Posterior facial height (mm)	Female	75.11±5.11	76.40±4.76	76.26±4.75	73.37±5.17	< 0.001**	1, 2 > 3
	Male	84.18±6.36	86.01±5.82	82.72±5.90	82.49±6.97	0.047*	NS
Facial height index (%)	Female	66.21±4.66	67.48±4.49	67.31±4.53	64.53±4.39	< 0.001**	1, 2 > 3
	Male	69.75±5.68	71.38±5.25	69.11±4.61	67.72±6.48	0.034*	1 > 3
Y axis angle (°)	Female	61.02±3.47	61.34±3.39	60.58±3.49	61.23±3.48	0.366	
	Male	61.22±3.95	61.13±3.60	59.38±3.52	62.84±4.24	0.012*	2 < 3
Palatal plane angle (°)	Female	-0.13±2.66	-0.56±2.95	-0.13±2.55	0.11±2.59	0.355	
	Male	0.37±2.85	0.27±2.73	0.26±3.21	0.62±2.82	0.871	
Mandibular plane angle (FMA) (°)	Female	24.67±5.34	23.84±5.56	23.76±5.39	25.94±4.96	0.013*	1, 2 < 3
	Male	22.97±6.78	21.65±6.65	22.39±5.57	25.52±7.38	0.074	
Occlusal plane angle (°)	Female	8.42±3.86	8.42±3.76	7.99±3.61	8.80±4.12	0.398	
	Male	8.02±4.83	7.43±4.15	6.76±4.34	9.95±5.72	0.050	2 < 3
3. Sagittal Relationship							
SNA (°)	Female	82.56±3.38	82.53±3.32	83.07±3.19	82.12±3.55	0.185	
	Male	83.20±3.96	84.52±3.83	83.15±3.52	81.18±3.75	0.003**	1 > 3
SNB (°)	Female	77.98±3.74	78.03±3.74	78.92±3.52	77.13±3.76	0.007**	1, 2 > 3
	Male	79.21±4.47	80.19±4.51	80.30±3.91	76.82±4.03	0.005**	1, 2 > 3
ANB (°)	Female	4.58±2.71	4.49±2.31	4.16±2.99	4.99±2.63	0.134	
	Male	3.99±3.32	4.34±3.30	2.85±2.61	4.37±3.74	0.212	
Wits (mm)	Female	0.49±3.63	0.63±2.99	0.13±3.88	0.74±3.76	0.523	
	Male	0.93±4.42	0.93±4.41	-0.60±3.84	0.80±5.14	0.445	
4. Mandible position and morphology							
Mandibular arc angle (°)	Female	37.14±5.22	38.48±5.42	37.82±4.77	35.79±5.22	0.004**	1, 2 > 3
	Male	38.06±5.67	39.00±5.42	36.92±4.51	37.53±6.76	0.354	
Mandibular body length (mm)	Female	68.34±4.25	68.22±3.61	69.05±4.47	67.78±4.34	0.150	
	Male	72.90±5.39	72.93±5.29	73.82±3.92	72.10±6.54	0.573	
Ramus height (mm)	Female	45.21±3.97	46.24±3.69	45.93±3.72	44.00±4.04	0.001**	1, 2 > 3
	Male	51.00±5.09	52.38±4.85	49.97±5.22	49.68±5.00	0.067	
Articular angle (°)	Female	151.69±6.85	150.58±6.85	151.49±5.94	152.50±7.55	0.264	
	Male	149.92±6.84	149.45±6.85	149.15±7.10	151.27±6.70	0.500	
Gonial angle (°)	Female	117.99±6.08	116.85±5.65	117.49±5.98	119.07±6.29	0.076	
	Male	116.26±7.13	115.25±7.71	117.94±6.23	116.26±7.13	0.391	
5. Dental Relationship							
Interincisal Angle (°)	Female	126.85±13.16	126.44±14.19	126.72±14.15	127.20±11.72	0.942	
	Male	128.28±13.34	126.49±12.41	128.61±14.27	130.79±14.07	0.455	
IMPA (°)	Female	96.39±7.87	97.73±7.77	96.88±8.61	95.19±7.14	0.144	
	Male	97.57±9.34	100.07±8.43	95.17±9.38	95.61±10.02	0.072	
FMIA (°)	Female	58.95±9.13	58.45±8.60	59.36±9.90	58.87±8.80	0.854	
	Male	59.46±9.86	58.30±7.90	62.46±9.92	58.86±12.23	0.292	

Overbite (mm)	Female	2.51±2.00	2.51±2.15	2.59±1.76	2.43±2.14	0.875
	Male	2.75±2.72	2.65±2.60	2.25±2.05	3.29±3.31	0.428
Overjet (mm)	Female	4.10±1.79	4.16±2.16	3.88±1.72	4.26±1.60	0.362
	Male	3.82±2.30	3.77±2.62	3.40±2.09	4.23±1.90	0.481

Data presented by mean ± SD; NS no significance, * P value<0.05; ** P value<0.01.

Table S2. Stratified analysis based on sagittal skeletal pattern

		Overall	Normal	DDwR	DDwoR	*P* value	Post-hoc
**1. Cranial Base**							
Anterior cranial base length (mm)	Class I	63.06±3.51	63.54±3.43	63.47±3.63	62.12±3.33	0.077	
	Class II	62.26±3.11	62.92±3.30	61.91±2.61	62.09±3.28	0.349	
	Class III	63.43±2.80	62.50±3.39	63.32±2.20	64.51±2.97	0.364	
Posterior cranial base length (mm)	Class I	33.83±3.36	34.65±3.37	33.66±2.99	33.18±3.61	0.088	
	Class II	32.82±3.50	34.05±4.15	33.12±2.45	31.95±3.47	0.018*	1 > 3
	Class III	33.83±3.85	36.19±3.20	33.28±3.97	32.31±3.55	0.103	
Saddle angle (°)	Class I	124.84±4.87	125.26±5.66	123.76±4.59	125.56±4.15	0.143	
	Class II	125.05±5.58	125.10±5.91	125.47±5.00	124.77±5.79	0.839	
	Class III	123.69±5.61	125.74±5.65	120.68±4.78	126.16±5.13	0.042*	NS
**2. Vertical relationship**							
Anterior facial height (mm)	Class I	115.34±6.89	116.04±6.49	115.06±7.34	114.92±6.89	0.684	
	Class II	116.18±6.53	117.76±6.88	115.21±6.53	115.89±6.28	0.242	
	Class III	114.55±8.20	114.59±7.09	112.53±9.26	117.56±7.55	0.420	
Posterior facial height (mm)	Class I	78.77±6.68	80.97±7.05	78.41±5.61	76.90±6.84	0.009**	1 > 3
	Class II	76.08±6.44	79.49±7.02	76.43±4.56	73.95±6.31	< 0.001**	1 > 2, 3
	Class III	78.55±7.99	82.51±7.76	77.34±8.01	76.41±7.74	0.253	
Facial height index (%)	Class I	68.34±4.78	69.80±4.94	68.25±4.36	66.94±4.70	0.012*	1 > 3
	Class II	65.54±0.50	67.52±4.66	66.48±4.62	63.86±4.94	0.001**	1, 2 > 3
	Class III	68.67±6.26	72.15±7.07	68.78±5.04	65.04±5.68	0.070	
Y axis angle (°)	Class I	60.29±3.24	60.61±3.34	59.87±3.25	60.42±3.13	0.502	
	Class II	62.64±3.37	62.55±3.22	61.93±3.26	63.12±3.49	0.244	
	Class III	58.08±3.27	59.79±3.97	57.54±3.22	57.16±2.08	0.213	
Palatal plane angle (°)	Class I	-0.09±2.68	-0.21±2.88	-0.12±2.58	0.06±2.63	0.880	
	Class II	0.10±2.69	-0.29±2.57	-0.31±2.86	0.57±2.61	0.188	
	Class III	0.09±3.13	0.20±4.16	1.01±2.54	-1.39±2.52	0.250	
Mandibular plane angle (FMA) (°)	Class I	22.81±5.39	21.98±6.11	22.74±5.19	23.75±4.72	0.268	
	Class II	26.26±5.69	24.62±5.30	25.06±5.75	27.92±5.50	0.008**	1, 2 < 3
	Class III	22.09±5.80	21.33±8.28	21.90±4.62	23.15±5.07	0.822	
Occlusal plane angle (°)	Class I	7.52±4.05	7.61±4.28	7.45±3.73	7.50±4.22	0.980	
	Class II	9.63±3.95	8.98±2.96	8.66±3.79	10.59±4.35	0.037*	NS
	Class III	6.45±3.85	6.26±4.81	6.21±3.55	7.01±3.69	0.895	
**3. Sagittal Relationship**							
SNA (°)	Class I	82.33±3.45	82.92±3.73	82.51±3.30	81.55±3.25	0.134	
	Class II	83.59±3.51	84.38±3.58	84.32±2.93	82.71±3.64	0.020*	NS
	Class III	80.93±3.34	82.20±3.07	81.86±2.95	78.26±2.93	0.021*	1, 2 > 3
SNB (°)	Class I	79.04±3.51	79.37±3.96	79.41±3.22	78.32±3.26	0.223	
	Class II	76.64±3.71	77.40±3.83	77.51±3.05	75.68±3.84	0.025*	NS
	Class III	82.23±3.77	82.95±4.47	83.38±3.39	79.76±2.61	0.084	
ANB (°)	Class I	3.29±1.09	3.54±1.14	3.11±1.00	3.23±1.11	0.125	
	Class II	6.96±1.62	6.99±1.90	6.81±1.56	7.04±1.51	0.794	
	Class III	-1.30±1.92	-0.74±2.21	-1.53±1.58	-1.54±2.21	0.631	
Wits (mm)	Class I	-0.43±2.76	0.08±2.85	-0.70±2.54	-0.50±2.90	0.518	
	Class II	2.99±2.98	3.28±3.13	2.94±2.74	2.86±3.08	0.811	
	Class III	-5.93±3.02	-4.50±1.74	-6.02±3.06	-7.24±3.62	0.195	
**4. Mandible position and morphology**							
Mandibular arc angle (°)	Class I	38.20±5.60	39.20±5.40	38.02±5.09	37.36±6.23	0.263	
	Class II	36.34±4.94	37.86±5.42	37.27±3.88	34.92±4.91	0.009**	1, 2 > 3
	Class III	38.01±5.21	39.20±5.51	37.10±5.53	38.19±4.83	0.689	
Mandibular body length (mm)	Class I	70.59±4.95	70.94±4.66	70.89±4.68	69.92±5.54	0.521	
	Class II	67.80±4.46	68.98±5.11	67.88±4.26	67.09±4.11	0.148	
	Class III	72.54±5.10	71.39±5.89	72.66±4.29	73.53±5.84	0.716	
	Class I	47.70±4.91	49.32±5.47	47.37±4.33	46.40±4.52	0.011*	1 > 2, 3

Ramus height (mm)	Class II	45.53±4.62	47.87±4.33	45.86±3.84	44.02±4.68	< 0.001**	1 > 2, 3
	Class III	47.96±6.14	50.58±6.60	46.76±5.71	47.15±6.28	0.372	
Articular angle (°)	Class I	150.32±6.23	149.46±5.59	151.08±6.49	150.39±6.58	0.429	
	Class II	152.98±7.17	152.54±7.46	151.09±5.90	154.38±7.52	0.087	
	Class III	147.72±6.86	143.86±7.00	150.50±6.52	147.40±5.95	0.102	
Gonial angle (°)	Class I	116.76±6.55	115.73±6.54	116.86±6.27	117.70±6.84	0.334	
	Class II	118.05±6.13	116.32±6.51	118.31±5.51	118.86±6.18	0.155	
	Class III	119.02±6.78	118.15±8.12	118.45±6.43	120.75±6.44	0.707	
**5. Dental Relationship**							
Interincisal Angle (°)	Class I	128.50±12.99	128.60±12.93	127.37±13.60	129.61±12.55	0.694	
	Class II	124.11±13.00	122.26±13.63	124.09±14.46	125.15±11.75	0.596	
	Class III	134.95±11.22	130.69±11.97	135.03±13.12	139.09±5.62	0.338	
IMPA (°)	Class I	95.53±7.43	97.37±8.07	95.89±7.74	93.27±5.78	0.021*	1 > 3
	Class II	100.29±6.96	102.34±6.18	100.91±7.51	98.77±6.78	0.049*	NS
	Class III	86.61±8.44	92.25±9.49	86.18±6.99	81.64±6.59	0.034*	1 > 3
FMIA (°)	Class I	61.66±7.95	60.67±7.30	61.38±8.90	62.98±7.48	0.344	
	Class II	53.44±7.15	53.05±6.51	54.02±7.91	53.31±7.11	0.839	
	Class III	71.30±7.03	66.43±7.98	71.94±6.19	75.21±4.60	0.033*	1 < 3
Overbite (mm)	Class I	2.58±2.20	2.57±2.18	2.28±1.58	2.91±2.72	0.364	
	Class II	2.80±2.15	2.93±2.29	2.94±2.04	2.63±2.16	0.730	
	Class III	1.50±2.42	1.11±3.17	2.27±1.99	0.74±2.12	0.345	
Overjet (mm)	Class I	3.87±1.44	3.81±1.74	3.74±1.22	4.07±1.32	0.487	
	Class II	4.68±1.92	4.95±2.47	4.53±1.67	4.62±1.72	0.628	
	Class III	1.84±2.56	1.16±2.94	1.71±2.61	2.70±2.12	0.490	

Data presented by mean ± SD; NS no significance, * P value<0.05; ** P value<0.01.
